# The Joys of Misery

**Published:** 1875-10

**Authors:** Ben. H. Pratt


### THE BISTOURY.

Vol. XI. ELMIRA, N. Y., OCT., 1875. No. 3.

### Contributions.

### THE JOYS OF MISERY.

BEN. H. PRATT.

The idiosyncrasies of men are riddles far more difficult of solution than that of the fabled Sphinx. To the man who observes effects philosophically, and would trace these effects back to a natural, sufficient and reasonable cause; to one to whom effects that cannot be so traced are a source of discomfort, to say the least; the peculiarities of some men are a misery. He who is blessed with health and strength and a modicum of this world's possessions; who rises refreshed and begins his daily toil with a glad heart and a face that cheers all whom he meets; who has a pleasant "good morning" for all, and carries the sunshine of life into every company he greets; who is jolly under circumstances which would depress the feelings of most men; this man is no enigma. We see at once the sources of his mirth—he is well, and he rejoices in his integrity of physique. He has such an excellent cause for his cheerfulness that we do not presume to ask him why he is happy.

Even the man to whom nature has not vouchsafed a normal condition of health—who has weaknesses, and ailments, and trials, of which few but himself are cognizant—who strives to conceal his infirmities, not because he is ashamed of them, but lest, by publishing them, he prove a leaven of misery which shall vitiate the multitude with which he comes in contact; this man is no enigma, for it is natural that men should strive to make their fellows happy rather than unhappy. The out-leaping joy of the jolly man, and the suppressed ailments of the afflicted man, are both readily explained upon the admitted principle of being, that the normal condition of society is mirthfulness, and the aim of each integer of the great body social is to sustain that condition to the best of his ability—to create mirth, if he can—to sustain that already created—at least not to make men unhappy without a good and sufficient cause.

But who can explain the mystery of the joys of misery? Who is there that has so carefully analyzed the dispositions of men that he can account for the enjoyment they take in being and making miserable? Varied as are the peculiarities of those with whom we come in daily contact, there is always seen some excuse for such, except in the case of those who take delight in their own infirmities, and are continually impressed with an unfortunate and ineradicable conviction, that inasmuch as they themselves are only happy when dwelling upon their own ills, others must be just as happy in listening to the narration thereof. Indeed, such seem to think that they are conferring a great boon upon their fellow men, for as the joys of misery are only vouchsafed to a few, these seem to think that they must describe them as minutely as possible, inasmuch as they cannot transmit the sublime ecstacy of the pains themselves.

We are prone to ridicule the expression "enjoying poor health"—and when we analyze the reasons why we ridicule it, we find that we have considered the phrase a misnomer, since, at the first impression, we would naturally assert that one could not enjoy pain. A close observation of the characteristics of many people, however, has convinced us that, with numbers of men and women, pain is pleasure, misery is joy, invalidism is a pursuit, and that more genuine enjoyment is derived from nursing their ailments, real and imaginary, and proclaiming them upon all occasions, than are gleaned from any of the other sources which nature and art have spread before them.

Of all the anomalies of our nature, this seems the most absurd and the most unaccountable. That most of these people are sincere in believing themselves specially favored in being the objects of sympathy for a whole neighborhood, is too plain to be denied; and yet we are not sufficiently uncharitable to believe that it is for the sake of eliciting sympathy that they persist in proclaiming their ills. There are those who have a morbid itching for sympathy, but this desire seldom carries them so far as to make them the objects of avoidance; and there are none who are so fond of sympathy that they will make themselves wretched in order to secure it. Hence we are in a quandary, not as to the motive of the communicative hypochondriac, but as to the hidden causes within his nature which drive him out into the world, a peripatetic dispenser of miseries.

The catalogue of diseases which one of these unenviable mortals will regale a companion with, would entitle the relater to a medical diploma. Whatever the afflictions he may be actually possessed of, he will detail a hundred or more collateral diseases—side issues, as it were—reserves to fall back upon in case he should be deprived of some one of his favorite maladies. There is no organ in his entire system that exists entire, or sustains a normal action. He will prove to you, beyond a shadow of a doubt, or the possibility of a contradiction, that he is a living miracle of humanity—one who ought, in the ordinary course of hygienic events, to have been in his grave years ago; one whose sufferings have been and still are so far beyond his ability to tell, that he fairly agonizes over his lack of words—words strong enough to represent his tortures. These he will relate to you with a volubility that would seem to contradict his assertions of infirmity. His lungs, if you will believe him, are but shriveled apologies for the healthy organs which his vocal efforts would make you think they are. His stomach has none of the characteristics of other stomachs, and yet he manages to make it do receptacle duty in a manner that would surprise a wood-chopper. His heart is eternally upon its final flutter, and still that last crisis don't seem to ensue, and the pulse don't indicate that it's likely to. His liver is indurated or degenerated, or enlarged, or ossified or something worse, and he lives a perpetual monument of miraculous vitality. Of the rheumatisms and neuralgias he's endured, there's no end to the recital. In a word, from the sole of his foot to the summit of his cranium, there isn't a healthy fibre in his entire anatomy. He entertains no propositions of profit or pleasure, for there is nothing so attractive to him as the prospect of discovering a new malady within himself, and naught is so enjoyable in his eyes as an opportunity to breathe the result of the same into a listening ear, or to rehearse it unto a crowd of sympathizing auditors.

But our object at present is not so much to acquaint the reader with the fact that there are such people as we have referred to above, for it would be a singular fact if there were a single adult individual who has not encountered one or more of these social plagues; nor yet in the hope of persuading these misery lovers to abandon the practice of giving such wide publicity to their precious woes, for this would deprive them of about the only enjoyment which society affords them. It is only to say to this class of invalids, that we who are well, and also we whose maladies are sacred, being admitted by ourselves and to ourselves, only under protest, and communicated only to our doctors and those who have a right to a knowledge of that which concerns our welfare; to say, that this eternal din of exaggerated infirmity, in season and out of season—in our walks to and from business—in our social chats—at the table—everywhere—is a bore of the very worst description, and it is time that these misery makers learn that if they are sick, they are entitled to, and have, our heartfelt sympathies, and that any service we can render them will be gladly and willingly performed; that our memories are a fair average, and do not require a reiteration of the dismal catalogue of woes which these people carry at their tongues' end and insist upon retailing to us every time we meet them; that if they are determined to so inflict their trials upon us, we must claim the privilege of letting them know that we don't enjoy that sort of thing, and have set them down as bores of the first water, and shall avoid them upon every possible occasion. This may seem a harsh and heartless method of treatment for sick folks, but they should remember that the well have rights which the sick are bound to respect, and that in the busy round of active life, they who cannot participate in its activity should keep out of the way in order that the well may, by dint of hard labor and rough knocks, do the work which is abandoned by those who apparently enjoy being sick, and in which work no assistance is rendered by those who are detailed for attendance upon the hypochondriacal.

Again, we say, that the world is apt to regard him who is eternally complaining, either as an impostor—one who is too lazy to take his part in the rough and tumble of life; or a lunatic—one whom the community deems best off in his own chamber or within the walls of an asylum, where he can enjoy his precious infirmities without infusing an *ennui* through an entire neighborhood. The world wants such folks to know precisely in what estimation they are held, and to give that information is the object of this article.

---

KEEP THEM.—The BISTOURY will be printed in such a manner as to exclude advertising sheets in binding. Therefore preserve all the numbers.

